# Vascular gene expression: a hypothesis

**DOI:** 10.3389/fpls.2013.00261

**Published:** 2013-07-17

**Authors:** Angélica C. Martínez-Navarro, Santiago V. Galván-Gordillo, Beatriz Xoconostle-Cázares, Roberto Ruiz-Medrano

**Affiliations:** Department of Biotechnology and Bioengineering, CINVESTAV-IPNMexico DF, Mexico

**Keywords:** vascular tissue, phloem, evolution, companion cell, sieve element

## Abstract

The phloem is the conduit through which photoassimilates are distributed from autotrophic to heterotrophic tissues and is involved in the distribution of signaling molecules that coordinate plant growth and responses to the environment. Phloem function depends on the coordinate expression of a large array of genes. We have previously identified conserved motifs in upstream regions of the Arabidopsis genes, encoding the homologs of pumpkin phloem sap mRNAs, displaying expression in vascular tissues. This tissue-specific expression in Arabidopsis is predicted by the overrepresentation of GA/CT-rich motifs in gene promoters. In this work we have searched for common motifs in upstream regions of the homologous genes from plants considered to possess a “primitive” vascular tissue (a lycophyte), as well as from others that lack a true vascular tissue (a bryophyte), and finally from chlorophytes. Both lycophyte and bryophyte display motifs similar to those found in Arabidopsis with a significantly low *E*-value, while the chlorophytes showed either a different conserved motif or no conserved motif at all. These results suggest that these same genes are expressed coordinately in non-vascular plants; this coordinate expression may have been one of the prerequisites for the development of conducting tissues in plants. We have also analyzed the phylogeny of conserved proteins that may be involved in phloem function and development. The presence of CmPP16, APL, FT, and YDA in chlorophytes suggests the recruitment of ancient regulatory networks for the development of the vascular tissue during evolution while OPS is a novel protein specific to vascular plants.

## Introduction

The vascular tissue played an essential role in the adaptation of plants to land, allowing the great diversification of tracheophytes, plants harboring conducting tissues. While non-vascular land plants are also quite diverse and include species that have successfully colonized a variety of niches, the sheer number of tracheophyte species as well as its diversity in geographical distribution, shape and size is overwhelming. This in no small part has resulted from this evolutionary innovation.

The evolution of specialized cell types that gave rise to conducting tissues allowed the distribution of nutrients to all plant organs, which evidently also led to cell and tissue specialization, or, as is also termed, a division of labor. The vascular tissue gave rise more recently to two well-defined transport systems within plants, the xylem and the phloem. While the first one has a direct role in the delivery of water and mineral nutrients from roots to aerial tissues, the phloem is involved in the transport of fixed carbon as well as other nutrients from photosynthetic to heterotrophic tissues. In addition, a wealth of evidence indicates that the vasculature functions in signaling between distant tissues (Lucas et al., 2013). Indeed, on one hand, several responses to environmental cues, as well as to a genetic program, imply such long-distance signaling. For example, response to drought stress is regulated by abscisic acid (ABA) transport from roots to shoots via the xylem (Sauter et al., 2001); the control of nodule number in legumes results from the transport of a signaling molecule from leaves to roots, presumably through the phloem (Krusell et al., 2002). Flower induction, the paradigm of phloem inter-organ signaling, which has been extensively reviewed (Corbesier et al., 2007; Lin et al., 2007; Tamaki et al., 2007), involves the transport from photosynthetic leaves to the shoot apex of a protein, FLOWERING LOCUS T (FT; and possibly also RNA). Thus, it is clear that the vascular tissue, and in particular the phloem, the focus of this work, plays an essential role in plant adaptation.

Plants include the tracheophytes, chlorophytes, (single-celled and colonial taxa) and bryophytes. Although obviously the extant representatives of chlorophytes and non-vascular bryophytes cannot by any means be considered primitive or less evolved than vascular plants, it can be argued that they share some primitive features common to all plants ancestors, such as unicellularity or less modified cell types. These can also illustrate the likely steps (not necessarily in chronological order, and most probably occurring simultaneously and evolving independently in different plant lineages) that gave rise to modern vascular plants, evidently starting with the evolution of multicellularity. Next, specialization occurred in such manner that originated heterotrophic cells, including those that were able to absorb mineral nutrients and water from soil as well as those that gave rise to reproductive tissue (Lucas et al., 2013). It can be envisaged that other events in the early evolution of land plants involved the establishment of novel developmental programs that resulted in sieve cells (as in gymnosperms) and eventually in sieve elements on one hand, and in vessel elements on the other. The genetic networks underlying such processes have been intensely studied, more so in the case of xylem differentiation, in which case a study using comparative genomics revealed that xylem transcriptomes were more conserved during evolution than other tissues transcriptomes in vascular plants, being the functional domains of genes specially conserved pointing to the presence of an ancestral xylem transcriptome; also, a phylogenetic analysis showed that evolution of xylem transcriptome follows the branch divergence patterning than plant species (Li et al., 2010). Less is known on the pathways leading to Companion Cell-Sieve Element (CC-SE) differentiation, although recent work has helped identify key proteins involved in such process.

Efforts have been made to identify genes involved in the transition from unicellular algae to multicellular land plants as well as the transition from non-vascular to vascular plants, uncovering that most of the genes involved in vasculature formation and differentiation were already present in non-vascular plants; so the evolution of vasculature involved, additionally to some innovations, the co-option and integration of ancestral developmental pathways (Banks et al., 2011).

It is likely that reprogramming the expression of certain genes must have been important during the evolution of the vasculature. In particular, it is conceivable that the evolution and development of new structures or tissues in any organism necessarily involves the coordinated expression of a set of genes, both temporally and spatially.

In the present work, we addressed the evolution of vascular gene expression by examining regulatory upstream regions of genes from various species, the homologs of which in pumpkin encode transcripts found in pumpkin phloem sap exudates, ranging from chlorophytes to monocots and dicots, but also including a plant lacking true vascular tissue, and another one that displays “primitive” conducting tissues. We selected these genes that have the highest number of ESTs found in phloem sap exudates, although it is clear that these may be expressed in other tissues in addition to phloem. Furthermore, the upstream regions of these genes from Arabidopsis (termed SETP, for Sieve Element Transcript Promoters set) harbor an overrepresented common motif, directing gene expression in the vasculature in mature leaves (Ruiz-Medrano et al., 2011). It must be mentioned that several of the analyzed genes from Arabidopsis have not been characterized in detail and thus the transcriptional start site is not known. The present work is based on some assumptions: (1) Not all phloem sap transcripts are necessarily transported through the phloem, since some may originate from neighboring damaged cells; however, it is likely that most are synthesized in phloem cambium, CC or phloem parenchyma and thus reflect gene activity in the phloem, or at least in vascular tissues, as evidenced by in situ hybridization of pumpkin phloem sap transcripts and histochemical analysis of plants expressing GUS fusions to upstream regions of the Arabidopsis gene homologs for these transcripts (Ruiz-Medrano et al., 1999a,b; Xoconostle-Cázares et al., 1999; Ruiz-Medrano et al., 2007, 2011). (2) Gene promoters harboring common motifs are expressed in a coordinated fashion; the more common motifs they share the coordination is tighter (spatially and/or temporally; see for example Harmer et al., 2000), although this should not be always necessarily the case. (3) The closest homolog to the corresponding pumpkin gene should be expressed in a similar fashion, i.e., will correspond to a gene also expressed in vascular tissue. This assumption is especially crucial when dealing with members of large gene families, not necessarily sharing a common expression pattern. (4) While the upstream regions of the analyzed genes are termed promoters, several have been poorly characterized and therefore may include 5′ untranslated regions (UTR). Therefore, it is possible that several motifs found in Arabidopsis lie within the 5′UTR of these genes (Ruiz-Medrano et al., 2011).

There is an implicit limitation in this approach, and it is that the analyzed sequences belong to taxa whose genomes have been deciphered. These taxa correspond to plants with basic and/or economic importance; thus, some of them may not be representative from an evolutionary viewpoint.

The predicted phylogeny of some key proteins involved in phloem function and differentiation was also analyzed. The results suggest that most dicot and some monocot genes expressed in vascular tissue share GA/CT motifs, as found in Arabidopsis (Ruiz-Medrano et al., 2011). Interestingly, no such motifs were found in chlorophytes, and at least in one of those analyzed, no common motif could be discerned in this gene promoter set. 51 SETPHs were found in chlorophytes but not in all analyzed algal species, suggesting an ancient function of these genes not related with vascular expression (Figure 5, Table 2) Finally, the dendrograms suggest that phloem evolution occurred by the recruitment of preexisting genes involved in certain regulatory networks, as well as the appearance of novel genes altogether.

## Materials and methods

The sequences analyzed are described in a previous work (Ruiz-Medrano et al., 2011). These correspond to genes for transcripts that have been found in pumpkin phloem sap exudates. It is reasonable to assume that the homologs from extant taxa are expressed in a similar manner, and that phloem sap transcripts result from the activity of gene promoters in the CC or neighboring cell types.

### Sequences

Upstream sequences from extant plant taxa were retrieved from phytozome (http://phytozome.net/). First, the protein sequences from Arabidopsis were obtained through the Arabidopsis Information Resource (TAIR; http://www.arabidopsis.org/). Afterwards, a BLAST search was carried out using the corresponding Arabidopsis protein sequence as query into the proteome database of interest; afterwards the upstream 1 kb sequences were obtained for each protein homolog from the data set (see Tables 1, 2, S1). 5′UTRs were included in case the transcriptional start site was not known for the gene in question.

**Table 1:** **List of SETPs used for this analysis**.

**ID**	**Function**	**Functional categorization**	**Tissue-specificity**
At1g04100	IAA10 indoleacetic acid-induced protein 10	Protein binding, transcription factor activity	Sperm cell, root protophloem and metaphloem protoplast, xylem, stem
At1g09060	Zinc finger, RING-type;Transcription factor jumonji/aspartyl beta-hydroxylase	Transcription factor activity	Sperm cell, shoot phloem companion cell
At1g16070	AtTLP8, TLP8 tubby like protein 8	Transcription factor activity	Shoot apex, root vascular tissue cell
At1g17440	EER4, TAF12B Transcription initiation factor TFIID subunit A	DNA or RNA binding	Chalazal seed coat, testa
At1g19210	Integrase-type DNA-binding superfamily protein	DNA or RNA binding, transcription factor activity	Root phloem pole pericycle protoplast, columella protoplast, xylem, chalazal endosperm
At1g19220	ARF19, IAA22, ARF11 auxin response factor 19	DNA or RNA binding, protein binding, transcription factor activity	Giant cell, xylem
At1g19600	pfkB-like carbohydrate kinase family protein	Kinase activity	Peripheral endosperm, hypocotyl, peripheral endosperm, hypocotyl
At1g34260	FAB1D FORMS APLOID AND BINUCLEATE CELLS 1A	Kinase activity, nucleotide binding, transferase activity	Sperm cell, pollen
At1g43700	VIP1, SUE3 VIRE2-interacting protein 1	DNA or RNA binding, protein binding, transcription factor activity	Chalazal seed coat, protoplast, giant cell
At1g43900	Protein phosphatase 2C family protein	Hydrolase activity	Shoot phloem companion cell, root phloem companion cell
At1g48090	Calcium-dependent lipid-binding family protein	Other binding	Sperm cell, guard cell
At1g49620	KRP7, ICN6, ICK5 cyclin-dependent kinase inhibitor family protein	Protein binding	Petal, columella protoplast, carpel
At1g51800	Leucine-rich repeat protein kinase family protein	Kinase activity, nucleotide binding, transferase activity	Leaf protoplast, root hair cell protoplast, guard cell, lateral root
At1g53300	TTL1 tetratricopetide-repeat thioredoxin-like 1	ND	Root xylem pole pericycle protoplast, root phloem pole pericycle protoplast, axillary root, pistil
At1g57700	Protein kinase superfamily protein	Kinase activity, nucleotide binding, transferase activity	Suspensor, testa
At1g61370	S-locus lectin protein kinase family protein	Kinase activity, nucleotide binding, transferase activity	Leaf protoplast, root phloem pole pericycle protoplast, pericycle, guard cell
At1g63700	EMB71, YDA, MAPKKK4 Protein kinase superfamily protein	Kinase activity, nucleotide binding, transferase activity	Chalazal seed coat, peripheral endosperm
At1g66150	TMK1 transmembrane kinase 1	Kinase activity, nucleotide binding, transferase activity, receptor binding activity	Root cortex protoplast, root endodermis and quiescent center protoplast, shoot apical meristem, leaf primordia
At1g77450	anac032, NAC032 NAC domain containing protein 32	DNA or RNA binding, transcription factor activity	Guard cell protoplast, root endodermis and quiescent center protoplast, pericycle, elongation zone
At1g79580	SMB, ANAC033 NAC (No Apical Meristem) domain transcriptional regulator	DNA or RNA binding, transcription factor activity	Columella protoplast, lateral root cap protoplast, root tip, interfascicular cambium cell
At1g80070	SUS2, EMB33, EMB177, EMB14 | Pre-mRNA-processing-splicing factor	ND	Endosperm, chalazal endosperm
At2g16750	Protein kinase protein with adenine nucleotide alpha hydrolases-like domain	Kinase activity, nucleotide binding, transferase activity	Stamen, root endodermis and quiescent center cell
At2g17290	CPK6, ATCDPK3, ATCPK6 Calcium-dependent protein kinase family protein	Kinase activity, nucleotide binding, protein binding, transferase activity	Pollen, root protoplast, senescent leaf, roots
At2g40270	Protein kinase family protein	Kinase activity, nucleotide binding, transferase activity	Leaf protoplast, senescent leaf, cauline leaf
At2g45950	ASK20, SK20 SKP1-like 20	Other enzyme activity	Sperm cell, root protophloem and metaphloem protoplast, pollen
At3g03770	Leucine-rich repeat protein kinase family protein	Kinase activity, nucleotide binding, transferase activity	Petal, chalazal seed coat
At3g07610	IBM1 Transcription factor jumonji (jmjC) domain-containing protein	Transcription factor activity	Giant cell, senescent leaf
At3g10550	MTM1, AtMTM1 Myotubularin-like phosphatases II superfamily	Hydrolase activity	Sperm cell, protoplast, suspensor
At3g14205	Phosphoinositide phosphatase family protein	Hydrolase activity	Pollen, root protoplast, guard cell
At3g15220	Protein kinase superfamily protein	Kinase activity, nucleotide binding, transferase activity	Starch sheath (endodermis) cell, root xylem protoplast, internode cell
At3g24240	Leucine-rich repeat receptor-like protein kinase family protein	Kinase activity, nucleotide binding, transferase activity	Lateral root primordium protoplast, root xylem pole pericycle protoplast, pericycle, root tip
At3g24550	ATPERK1, PERK1 proline extensin-like receptor kinase	Kinase activity, nucleotide binding, transferase activity	Chalazal seed coat, root cortex cell
At3g25840	Protein kinase superfamily protein	Kinase activity, nucleotide binding, transferase activity	Sperm cell, chalazal seed coat
At3g46290	HERK1 hercules receptor kinase 1	Kinase activity, nucleotide binding, transferase activity	Leaf protoplast, root cortex protoplast, stigma, radicle
At3g47570	Leucine-rich repeat protein kinase family protein	Kinase activity, nucleotide binding, transferase activity	Giant cell, xylem
At3g55470	CmPP16 homolog	Unknown molecular functions	Leaf protoplast, guard cell protoplast, root culture, senescent leaf
At3g55610	P5CS2 delta 1-pyrroline-5-carboxylate synthase 2	Kinase activity, transferase activity	Sperm cell, suspensor
At4g05420	DDB1A damaged DNA binding protein 1A	DNA or RNA binding, protein binding, nucleic acid binding	Xylem, cork
At4g11800	Calcineurin-like metallo-phosphoesterase superfamily protein	Hydrolase activity	Columella protoplast, root xylem pole pericycle protoplast, pericycle, stigma
At4g16360	5'-AMP-activated protein kinase beta-2 subunit protein	Kinase activity, protein binding, transferase activity	Stigma, abscission zone
At4g17880	Basic helix-loop-helix (bHLH) DNA-binding family protein	DNA or RNA binding, protein binding, transcription factor activity	Xylem, cork
At4g23900	Nucleoside diphosphate kinase family protein	Kinase activity, nucleotide binding, tranferase activity,	Root phloem protoplast, root protophloem and metaphloem protoplast, stamen, pedicel
At4g24890	ATPAP24, PAP24 purple acid phosphatase 24	Hydrolase activity	Root epidermal atrichoblast protoplast, stamen, phloem
At4g26690	SHV3, MRH5, GPDL2 PLC-like phosphodiesterase family protein	Kinase activity, hydrolase activity	Guard cell protoplast, root cortex protoplast, leaf primordia, petiole
At4g26930	MYB97, AtMYB97 myb domain protein 97	DNA or RNA binding, transcription factor activity	Pollen, phloem
At4g31630	Transcriptional factor B3 family protein	DNA or RNA binding, transcription factor activity	Starch sheath (endodermis) cell, internode cell
At5g02010	ATROPGEF7, ROPGEF7 RHO guanyl-nucleotide exchange factor 7	Other molecular functions	Root xylem protoplast, root stele protoplast, shoot apex, root vascular tissue cell
At5g03300	ADK2 adenosine kinase 2	Kinase activity	Root xylem pole pericycle protoplast, elongation zone, root tip
At5g03790	ATHB51, LMI1, HB51 homeobox 51	DNA or RNA binding, nucleic acid binding, transcription factor activity	Shoot apex, carpel
At5g07370	IPK2a, ATIPK2A inositol polyphosphate kinase 2 alpha	Kinase activity, transferase activity	Abscission zone, protoplast, cotylledon and leaf guard cell
At5g08630	DDT domain-containing protein	Unknown molecular functions	Sperm cell, chalazal endosperm
At5g47840	AMK2 adenosine monophosphate kinase	Kinase activity, nucleotide binding, transferase activity	Leaf primordia, shoot apex
At5g54380	THE1 protein kinase family protein	Kinase activity, nucleotide binding, transferase activity	Root cortex protoplast, root endodermis and quiescent center protoplast, elongation zone, cotyledon and leaf pavement cell
At5g64940	ATATH13, ATH13, ATOSA1, OSA1 ABC2 homolog 13	Transferase activity, transporter activity	Leaf primordia, shoot apex
At5g65210	TGA1 bZIP transcription factor family protein	DNA or RNA binding, protein binding, transcription factor activity	Leaf protoplast, radicle, roots
At5g66080	Protein phosphatase 2C family protein	Hydrolase activity	Root cortex protoplast, root epidermis and lateral root cap protoplast, petal, guard cell
At5g67380	CKA1, ATCKA1 casein kinase alpha 1	Kinase activity, nucleotide binding, protein binding, transferase activity	Columella protoplast, pollen, shoot apex

**Table 2:**
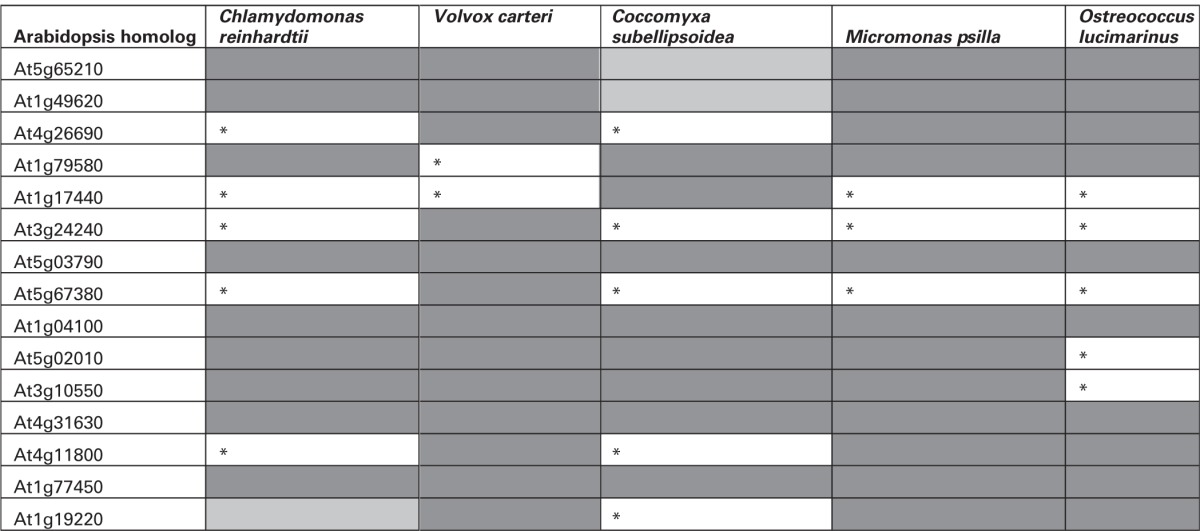
**List of Arabidopsis gene homologs not found in chlorophytes**.

^*^Present.

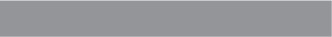
 *Not present*.

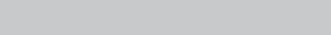
 *Potential homolog with a high E-value (>10^−3^)*.

### Gene list

The Functional categorization was obtained from TAIR. The tissue specific gene expression was obtained form GENEVESTIGATOR (www.genevestigator.com/).

### Promoter analysis

The upstream sequences were then compared with a probabilistic approach, using the Multiple EM for Motif Elicitation method (MEME; http://meme.nbcr.net/meme/; Bailey and Elkan, 1994) with the following settings: a maximum of 3 motifs per promoter, and 6–10 bases motif length. Also, the analyzed sequences were shuffled to provide for control sequences. The common motifs found for each promoter set were graphically represented using the Weblogo program (http://weblogo.threeplusone.com/create.cgi; Crooks et al., 2004).

### Phylogenetic analysis

Protein sequences for homologs of pumpkin CmPP16, and Arabidopsis FT, APL, Octopus and YDA were obtained from Phytozome. Protein sequences were retrieved from The Arabidopsis Information Resource database (www.arabidopsis.com). BLAST analysis was done in the Phytozome database (www.phytozome.net) and the most representative proteins in each taxa were selected. Aminoacid sequences were aligned using CLUSTAL X2 (Larkin et al., 2007) and edited manually with SEAVIEW4 program (Gouy et al., 2010). Maximum-likelihood and Neighbor-Joining phylogenies were generated form the alignments in MEGA5 (Tamura et al., 2011). The substitution matrix P-distance (Nei and Kumar, 2000) was used for Neighbor-joining and Dayhoff model (Schwarz and Dayhoff, 1979) for Maximum-likelihood reconstruction. Pairwise deletion Gaps/missing data treatment was chosen for Neighbor-Joining method and Partial deletion with a site coverage cutoff of 95% for Maximum-likelihood method. An heuristic search was used for Maximum-likelihood initial tree as follows: maximum parsimony method was used when the number of common sites was 100 or less than one fourth of the total number of sites; otherwise, the BIONJ method with MCL distance matrix was used. Replicates consisting in 1000 bootstraps were used for the statistical support of all phylogenetic trees.

## Results

### Most embryophytes share an overrepresented GA/CT motif in upstream regions of SETP homologs (SETPHs)

It was assumed that the closest homologs to genes expressed in vascular tissue in Arabidopsis would have a similar expression pattern in other species. Therefore, a BLASTP search was carried out for each of the plant species for which its genome has been sequenced. Then, the corresponding 1 kb upstream region was retrieved. The list of 57 Arabidopsis genes and their promoters, used to search for their homologs in other plant species is shownt These genes showed overlapping functions, according to their GO, of which 26 (45%) encoded kinases, 20 (35%) for nucleotide binding proteins and 14 (25%) for transcription factors; also, in most of them phloem from different organs is the tissue in which tgese genes are generally expressed at higher levels (data not shown), according to the Genevestigator database (Table 1). These were termed SETPH, based on the nomenclature described before, for Sieve Element Transcript gene Promoter Homolog (Ruiz-Medrano et al., 2011). There is information on the vascular expression of some of these genes, such as At1g34260, At1g63700, At1g14205, At5g65210, At1g19220, At3g03770, At3g55470, and At5g66080 (Ruiz-Medrano et al., 2011). Interestingly, in chlorophytes several genes were not found, or the similarity was too weak to consider these as orthologs of the Arabidopsis genes (Table 2).

**Figure 1:**
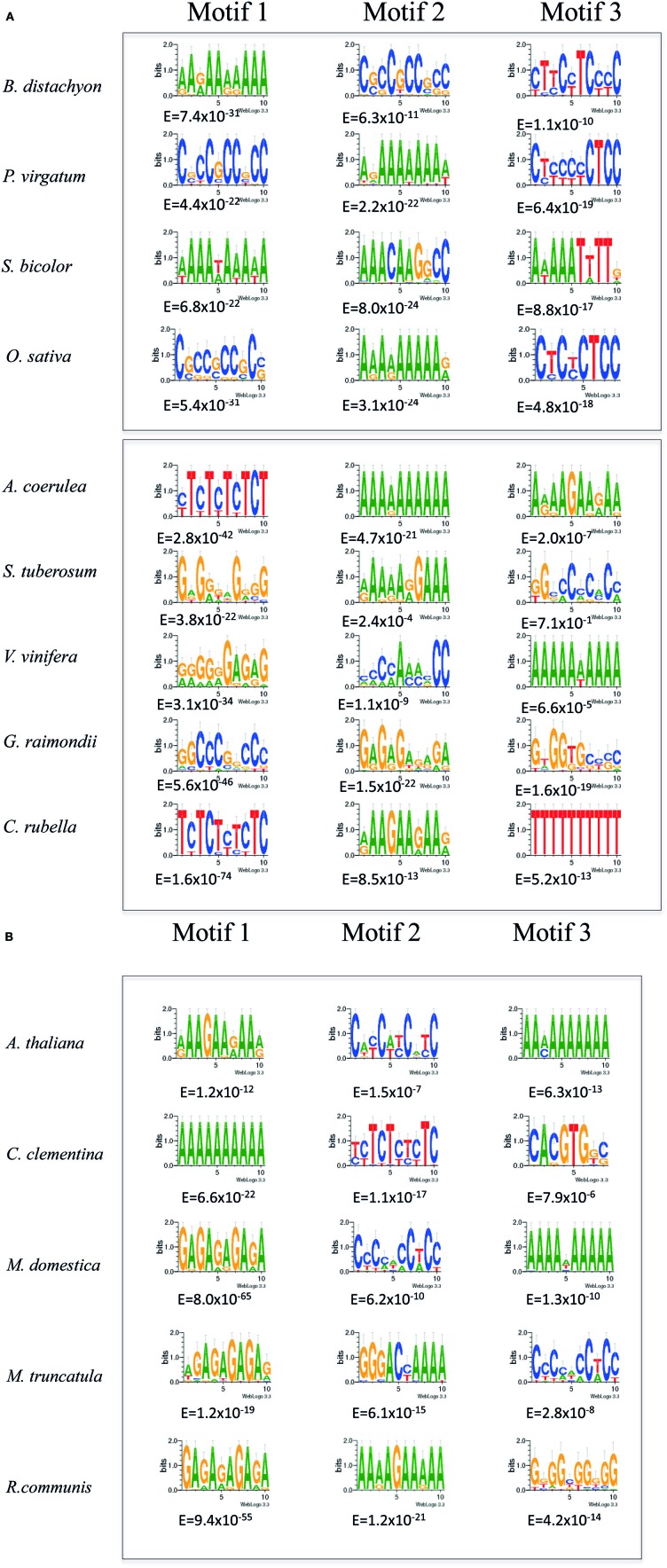
**(A,B)** MEME analysis of SETPHs from monocots (upper panel) and dicots (lower panel). Shown are the three overrepresented motifs in each promoter set identified by MEME analysis with the lowest Expectation (E) values below each motif.

A probabilistic method, Multiple EM for Motif Elicitation (Bailey and Elkan, 1994) has been used previously in our group in order to detect common motifs in upstream regions of Arabidopsis homologs of pumpkin genes for transcripts isolated from phloem sap exudates (Ruiz-Medrano et al., 2011). Indeed, this method predicted the most abundant motif in this promoter set, GA/CT repeats, which coincided with those found using enumerative methods. More importantly, promoters harboring such repeats were found to be active in vascular tissues, as well as in other tissues. Also recently, the CT rich motifs were reported in CRF (cytokinin response factors) family genes which have strongest expression in phloem tissue, supporting the notion that these motifs are conserved and overrepresented in promoters of genes expressed in this tissue (Zwack et al., 2012). Thus, this method was used for detection of common motifs, if any, in the same gene promoter set in other species. These are shown in Table S1, which includes most members of Viridiplantae, the genomes of which have been deciphered. These are mostly embryophytes, including the moss *Physcomitrella patens* as the sole representative of bryophytes; a tracheophyte with a less developed conducting tissue, *Selaginella moellendorffii*; and several dicot and monocot species. Also included are five species of chlorophytes showing colonial organization (*Volvox carteri*) as well as unicellular algae. The results of this analysis suggest that most dicots harbor a GA/CT rich motif similar to the Arabidopsis SETPs (Figures 1A,B). The three motifs with the lowest *E*-value are shown for each SETPH. In addition, common motifs were searched for in the same, shuffled sequences as a control. A cutoff value of *E* = 10^−6^ for Motif 1 was set; no common motifs were found in shuffled sequences whatsoever below this value. In the “native” sequences (i.e., not subjected to shuffling) those that did show such high values were present in few promoters and were quite degenerate. For example, in *Coccomyxa subellipsoidea* no motif with an *E*-value smaller than 1 was found, indeed, it was the only SETPH set in which no common motifs were found (Table S1).

In monocots, the grasses *Panicum virgatum* and *Brachypodium distachyon* SETPHs also harbored GA/CT rich motifs with a low *E*-value; a similar situation was found with *Setaria italica* (Table S1), suggesting that indeed the presence of the aforementioned motifs may correlate to vascular-specific expression. However, no such motif was found overrepresented in neither *Sorghum bicolor* nor *Zea mays* SETPHs; in this last instance relatively high *E*-values were found for the most overrepresented motifs, A/T-rich in both cases (Figure 1; Table S1).

**Figure 2:**
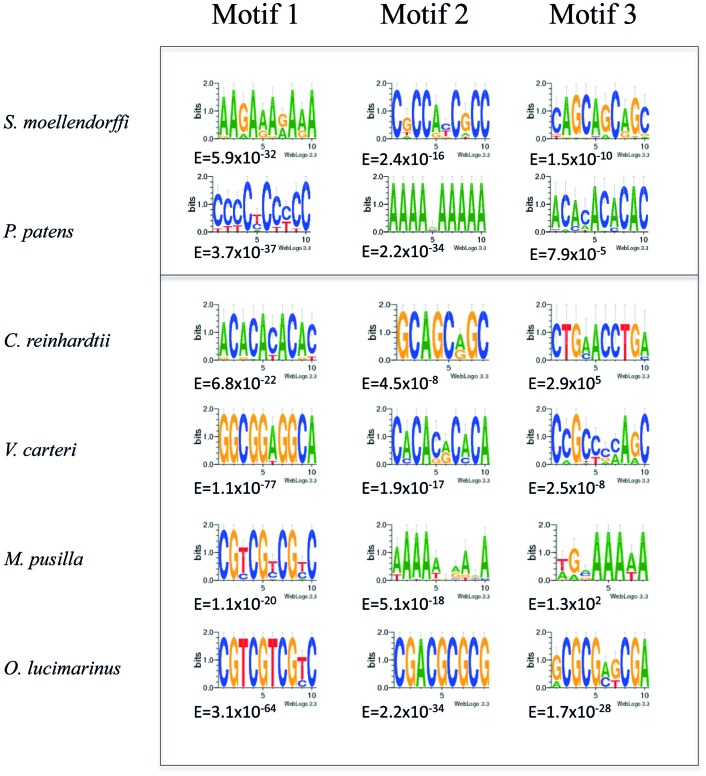
**MEME analysis of SETPHs from *S. moellendorffii* and *P. patens* (upper panel) and from chlorophytes (lower panel)**. Shown are the three overrepresented motifs in each promoter set identified by MEME analysis with the lowest Expectation (E) values below each motif.

Interestingly, a similar overrepresented motif was found in *S. moellendorffi* (Figure 2). This species is of particular interest because it has specialized, water-conducting tissues, albeit less complex than those observed in angiosperms and gymnosperms; in fact, the vessels of this plant have been considered as a “primitive” feature but involving the same character transformation as angiosperms and gymnosperms. Unlike angiosperms and gymnosperms, *Selaginella* phloem cells also present “primitive” features such as clustered pores as sieve areas and the presence of degenerated nuclei (Burr and Evert, 1973). Regardless, the presence of an overrepresented GA/CT rich motif in this SETPH with an extremely low *E*-value (10^−32^) supports the notion that these genes are expressed in a coordinated manner, and may be expressed also in its conducting tissues. On the other hand, C/G rich motifs with a low A/T content were detected in the *P. patens* SETPHs, which may be related to the GA/CT rich motif observed in tracheophyte SETPHs (Figure 2). It is worth mentioning that another overrepresented signature, a GT/AC–rich motif, was also present in some dicots; and, interestingly, in at least two chlorophyte SETPHs.

### Chlorophytes SETPHs share different common motifs

The same analysis was applied to the chlorophytes SETPHs in order to search for common motifs and determine whether these SETPHs may be expressed coordinately. Quite different motifs were found in this case; and, as mentioned before, one SETPH did not harbor common motifs, except for three shared by few promoters and of a degenerate nature (Figure 2; Table S1). It must be considered that no counterparts of 10–13 genes were found in chlorophytes (Table 2). While extremely low *E*-values were observed for some of these motifs (in particular a CGT repeat), these were present in a smaller subset of SETPHs, but at a high frequency in such promoters. Thus a more precise measurement of specific motif abundance was required in order to compare the frequency of such motifs in chlorophyte SETPHs as well as the GA/CT motif in embryophyte SETPHs.

### Chlorophytes and embryophytes SETPHs harbor distinct types of overrepresented motifs at different frequencies

A more quantitative analysis was carried out with the aforementioned SETPHs. First, the average number of each of the three motifs with lowest *E*-values per promoter was determined. Of mention, aside from the GA/CT and A/T rich motifs found in embryophytes, as well as the CGT and A/T rich motifs in chlorophytes, additional ones were found in other SETPHs. For example, a G-Box was found overrepresented in the *Citrus clementina* SETPH (Figure 1B). This *cis*-element is a transcription factor binding site involved in different types of responses, such as in light and drought (Liu et al., 2008; Lata and Prasad, 2011). On the other hand, a Conserved Late Element-like motif (CLE) was present in several *Medicago truncatula* SETPHs. This motif was first found in the Begomovirus common region, and is involved in late gene activation; interestingly, a small region of the Coat Protein gene promoter harboring these motifs is capable of directing vascular-specific expression (Sunter and Bisaro, 1997; Ruiz-Medrano et al., 1999b). Thus, vascular expression may require different motifs specific for each taxon. Additionally, the GA/CT rich motifs are also present in promoters active in tissues other than phloem, such as pollen, or in genes expressed in diverse tissues, such as ribosomal proteins and cyclins (Ruiz-Medrano et al., 2011).

**Figure 3:**
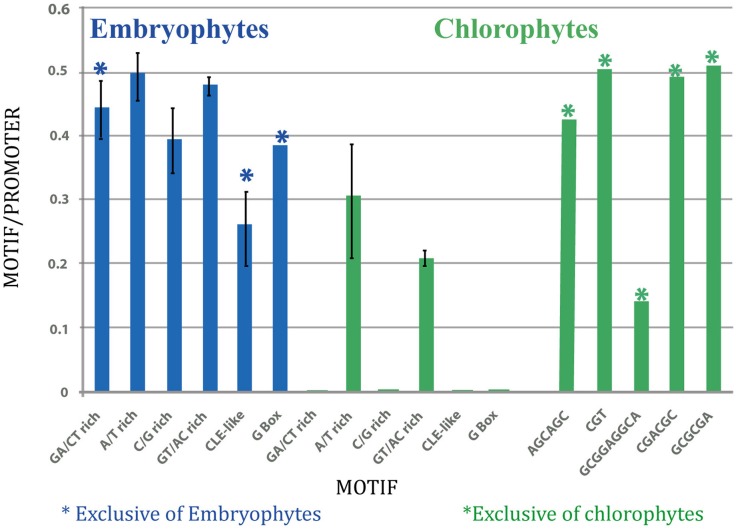
**Graphic representation of average number of motifs per promoter in embryophytes (left; blue), and in chlorophytes (right; green)**.

In the embryophytes SETPH, the motif with the highest average number per promoter was the A/T sequence, while in chlorophytes it was the CGT-repeat motif (Figure 3). However, this can be misleading, given that few promoters could harbor several of such motifs, thus biasing the abundance of such motifs in those particular SETPHs.

**Figure 4:**
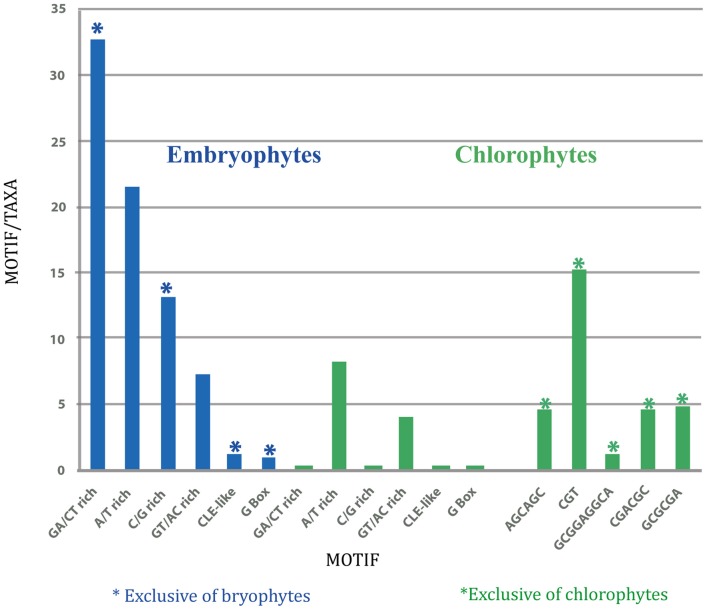
**Graphic representation of total number of motifs per total taxa in embryophytes (left; blue), and in chlorophytes (right; green)**.

Therefore, the number of each motif present per number of taxa was determined in order to estimate their relative abundance (Figure 4). Evidently, the number of GA/CT motifs was higher than all the other ones in embryophytes, while the CGT motif was more abundant in chlorophytes. Thus, the SETPHs of these two lineages could be expressed coordinately, except for *C. subellipsoidea*. However, the number of common motifs in chlorophytes is smaller than in embryophytes (Table S1), so a smaller subset of genes is expressed in a similar way. Nevertheless, *Ostreococcus* SETPHs showed the highest number of different common motifs in chlorophytes, suggesting that their expression is tightly coordinated (Table S1).

The results of this analysis suggest that the activity of the same set of SETPHs in different taxa from embryophytes is coordinated in a similar way, given the frequency of the shared GA/CT rich motifs. Interestingly, the same motif is overrepresented in *S. moellendorffii* and in *P. patens*, this last species lacking a true vascular tissue. Furthermore, a similar set of SETPHs in chlorophytes may also show some degree of coordination in its expression, suggesting that such potential coordination of gene activity was already present in ancient lineages leading to both chlorophytes and embryophytes. Furthermore, the absence of shared motifs in the SETPHs of *C. subellipsoidea* may suggest that this species is more distantly related from vascular plants than other chlorophytes.

### Phylogenetic analysis of proteins involved in phloem differentiation and/or function

From the previous section it can be inferred that coordinated expression of genes, aside from obvious functions in response to environmental stimuli or to a developmental program, may lead to novel structures, particularly in colonial or multicellular organisms. Evidently, proteins or other regulatory elements (such as non-coding RNAs) with novel features that may arose via gene or genome duplication, may had an important role in the evolution of multicellularity and in the development of new adaptation mechanisms (i.e., new developmental or signal transduction pathways, structures, etc.). The vascular tissue of plants is such an adaptation, while not necessarily a prerequisite for colonization of terrestrial ecosystems. Considering the diversity of non-vascular plants, it is evident that the vascular system represented an advantage that allowed the diversification and occupation of a large array of niches by tracheophytes, as well as a large increase in size. In particular, although the information regarding the factors involved in phloem differentiation and function is still fragmentary, recent work has discovered the role of some genes in phloem development (Lucas et al., 2013). The phylogeny of such genes, as well as those for factors that are presumably required for phloem function, should be helpful to determine the ontogeny of the vascular tissue during evolution.

The following proteins were selected for a phylogenetic analysis: (1) ALTERED PHLOEM DEVELOPMENT (APL). This protein is member of the MYB coiled-coil transcription factor family required for the proper formation of sieve elements and companion cells, and additionally inhibits xylem differentiation as demonstrated by analysis of *apl* mutants and ectopic expression of the gene (Bonke et al., 2003). (2) OCTOPUS (OPS) is a membrane-associated polar protein, which is expressed in provascular cells and later constrained to the phloem cell lineage, giving rise to the phloem cell continuity (Truernit et al., 2012). This gene is thought to act in a XYLOGEN-like manner although it does not possess any known domains required for its function (Truernit et al., 2012). (3). YDA, which is a MAPKK Kinase involved in asymmetric cell division during embryonary and stomatal development (Bergmann et al., 2004; Lukowitz et al., 2004). This protein positively regulates embryogenesis, while in stomata it inhibits stomatal precursor cell division. YDA is expressed also in vascular tissue, and more precisely in phloem (Ruiz-Medrano et al., 2011), so it is reasonable to assume that it has a role in phloem development. (4) Floral Locus T (FT). This protein, which binds phosphatidylethanolamine and is an inhibitor of RAF kinase, has a crucial role in flower induction; it is transported from mature photosynthetic leaves to the vegetative apical meristem where it induces flower initiation and thus is a major component of florigen (Kardailsky et al., 1999; Corbesier et al., 2007; Lin et al., 2007; Tamaki et al., 2007; Taoka et al., 2011). Thus, FT is involved in long-distance signaling, and would be expected to have acquired this novel function only in angiosperms. (5) CmPP16; this protein and its transcript were found originally in pumpkin phloem sap. CmPP16 has been shown to bind RNA in a non-selective manner and is able to assist its cell-to cell transport (Xoconostle-Cázares et al., 1999). Its immunolocalization, as well as the *in situ* localization of its RNA have demonstrated that both coincide in CC and in the phloem translocation stream, at least in cucurbits, and also discards the notion that these originate from damaged cells (Ruiz-Medrano et al., 1999a, 2007; Xoconostle-Cázares et al., 1999). Its role in phloem function is unknown, but its pervasive presence in all embryophytes to date suggests an important role in phloem function, likely in long- distance signaling.

All trees were constructed using sequences retrieved from the Phytozome database (www.phytozome.net). Figure S2 shows the resulting dendrogram for APL, using Maximum Likelihood. Three large clades are discernible: the tracheophytes, the lycophytes and bryophytes closely grouped together, and the chlorophytes. These do indeed harbor an APL homolog with a relatively low *E*-value ranging from 10^−14^ to 10^−16^, but showing in all cases similarity restricted to the N-terminus, which corresponds to the MYB DNA-binding domain (Prouse and Campbell, 2012). Thus a preexisting gene in the plant lineage may have been recruited for the phloem-specification program. This is a situation different from that of OCTOPUS. Indeed, no homologs for this protein were found in chlorophytes and *P. patens*. A sequence showing weak similarity, limited to 25 amino acids in its N-terminus, turned out in *S. moellendorffii* Thus, this sequence was utilized as outgroup. The results show that monocots and dicots form two separate clades (Figure S2). Interestingly, *Eucalyptus camaldulensis* OCTOPUS forms a single clade within tracheophytes equally distant to monocots and dicots. It is likely that this gene arose *de novo* during plant evolution, which reflects the relatively recent specialization of phloem development.

FT is a good example of a phloem-long distance signaling protein; it has distant homologs in bryophytes and lycophytes but not in chlorophytes. The maximum likelihood dendrogram shown in Figure S3 indicates that this gene is also a recent innovation, definitely present only in embryophytes. The *C. subellipsoidea* sequence was used as outgroup given its divergence. *S. moellendorffii* and *P. patens* form a single clade, and tracheophytes form another. However, the groupings observed within this clade do not correspond to the known phylogenetic relationships among vascular plants; for example, the *Vitis vinifera* FT homolog is the most divergent sequence within tracheophytes. Furthermore, there are four well-defined groups, one including monocots. Thus, the dendrogram may reflect functional specialization of FT rather than phyletic relationships between these plant taxa.

A Maximum Likelihood dendrogram of CmPP16 shows a topology not unlike FT (Figure S4). However, there are important differences. The first one, there is a clear potential homolog in a chlorophyte, *O. lucimarinus*, in contrast to other taxa from this group. This groups with the *P. patens* homolog. *C. subellipsoidea* “CmPP16” was again used as an outgroup; in general chlorophytes harbor sequences of very limited homology, if at all, and such similarity is restricted to the putative Cdomain found in the C-terminus of these proteins (*E*-value > 10^−3^). Additionally, the chlorophyte sequences are much larger (300–350 amino acids in length in chlorophytes vs. 130–150 amino acids in tracheophytes), and it is possible that the tracheophyte CmPP16 was derived from the aforementioned segment of an ancestral chlorophyte C2-domain containing protein. It must be mentioned that the Neighbor-Joining method failed to produce a meaningful dendrogram; the topology of this tree is essentially a large polytomy (Figure S1). This could be explained by high rates of substitution among these proteins.

The phylogenetic analysis of YDA suggests an ancient origin of this gene (Figure S5). *C. subellipsoidea* was also used as outgroup, given its weak similarity to embryophyte MAPKKK. In bryophytes and tracheophytes YDA homologs are numerous possibly by duplication events. Indeed, the pathways in which these proteins function allowed for the appearance of structures essential for the colonization of terrestrial (or more precisely, aerial) ecosystems, such as embryo, stomata, and vascular tissue (Hamel et al., 2006). The YDA homologs have a central conserved region that includes the serine/threonine kinase domain. Arabidopsis YDA possesses two unique domains, N-terminal and C-terminal, which greatly increases its size, possibly suggesting a functional specialization. The *Carica papaya* YDA homolog appears closely related to chlorophyte sequences perhaps because of its much smaller size than its angiopserm counterparts rather than for similarity.

## Discussion

### The origin of the vascular tissue may have involved gene coordination of ancestral SETPHs

The knowledge of the genetic networks that determine the identity and differentiation of vascular tissue is still rudimentary, although xylem ontogeny and evolution is better understood than that of phloem. Recent work has identified important genes directly related to phloem development; on the other hand, high throughoutput analysis of whole plant genomes, transcriptomes and proteomes provide tools for a more detailed evolutionary analysis of the vascular tissue. The present study aims to add some evolutionary perspective on the regulatory networks in which these genes function.

In a previous work we have identified a shared sequence signature in promoters of Arabidopsis genes expressed in vascular tissue (SETPs), a GA/CT-rich motif overrepresented in these upstream regions (Ruiz-Medrano et al., 2011). Thus, the same gene set was searched for common motifs in most plant genomes whose genome sequence is available (SETPHs). Similar motifs are found in all tracheophytes, and, interestingly, in the lycophyte *S. moellendorffii* and the bryophyte *P. patens*. The fact that most of these SETPHs harbor such motifs suggests that they are regulated coordinately in embryophytes, i.e., are expressed in vascular tissue. This is especially important considering, as has been mentioned before, that lycophytes and bryophytes lack a true vascular tissue, although *S. moellendorffii* as well as other lycopods do display water-conducting cells and thus could be considered an intermediate between land non-vascular and vascular plants (Niklas, 1997). Furthermore, given the diversity in development and morphology of these conducting cells, it has been suggested that the vascular system of plants arose more than once in the tracheophyte lineage (Niklas, 1997).

The most frequent motifs found in the embryophytes SETPHs, using the MEME algorithm, conform to the GA/CTn arrangement, or the closely related GAA/CTTn arrangement. In the first case it has been demonstrated that a GA octadinucleotide found in the promoter of a homeobox gene from barley regulates its transcription (Santi et al., 2003). A 126 bp fragment derived from the Arabidopsis *SUC2* gene promoter, harboring a GA/CT-rich sequence, was found to direct expression in the vasculature, and a short promoter fragment derived from a vascular expressed gene (and also harboring several GA/CT repeats), At1g34260 from Arabidopsis, was likewise capable of directing vascular expression when adjacent to the CaMV minimal promoter, although only consistently in T0 plants (Schneidereit et al., 2008; Ruiz-Medrano et al., 2011). In both cases the motifs present are similar, but not identical, to the auxin-response elements described previously (Li et al., 1994). This is in agreement with the fact that, while pervasive in embryophytes, the GA/CT motif was not identical between different SETPHs, and, in some cases, these were either not found or present at a high *E*-value. More importantly, auxin is a key regulator in vascular differentiation (Lucas et al., 2013). This could be explained considering that for various SETPHs, there is more than one homolog, so it is possible that the actual ortholog was not selected, or at least one that was not expressed in vascular tissue. It is noteworthy that a similar motif is found in non-vascular plants, with a very significant (low) *E*-value, and present in roughly half of the analyzed SETPHs (Table S1). It is then likely that the same set of genes that is expressed in vascular tissue in tracheophytes are also expressed coordinately in bryophytes. A different matter is what type of coordination do the expression of these genes display, temporal, spatial or both, but it is tempting to speculate that this coordination was important during the evolution of vascular tissue. It will be interesting to determine the expression patterns of these genes, now that the genetic transformation of these species is technically possible, which will surely shed light on the evolution of vascular gene expression.

As for chlorophytes, no GA/CT motifs were found in their SETPHs, but a CGT-repeat was found as the most frequent and with an extremely low *E*-value (Figure 4). However, these were concentrated in *O. lucimarinus* and *M. pusilla*, biasing its representation in these SETPHs. Nevertheless, it is worth mentioning that these genes, or most of them, may be expressed coordinately. Indeed, recent evidence indicates that in the case of *O. lucimarinus*, a marine picoalgae, there are several genes expressed in a coordinated fashion relative to genes from other microorganisms occupying the same niche, mostly in response to light (Ottesen et al., 2013). At least two of these genes are also present in the SETPH analyzed in this work, a calcium-dependent protein kinase and a MYB transcription factor family member. The frequency of common motifs with a significantly low *E*-value in *V. carteri* and *C. reinhardtii* is much lower, which could be interpreted as both having less genes from the SETPH set which are regulated coordinately. In any case, gene coordination in chlorophyte SETPHs may be not as extended as in embryophytes, and altogether absent in at least one case, *C. pseudoellipsoidea*. Evidently, the analysis of additional chlorophyte SETPHs as more genome sequences become available will allow determining the validity of these conclusions. It must be mentioned that vascular plants likely evolved from a streptophyte ancestor (Becker and Marin, 2009). Extant members of this group include multicellular green alga; unfortunately, to date no genomes from these taxa have been reported, but once available its analysis will afford valuable information regarding the evolution of the plant vasculature.

### Phylogenetic analysis of proteins involved in phloem differentiation and/or function support the notion that some arose *de novo* during evolution and others were recruited from preexisting pathways

**Figure 5:**
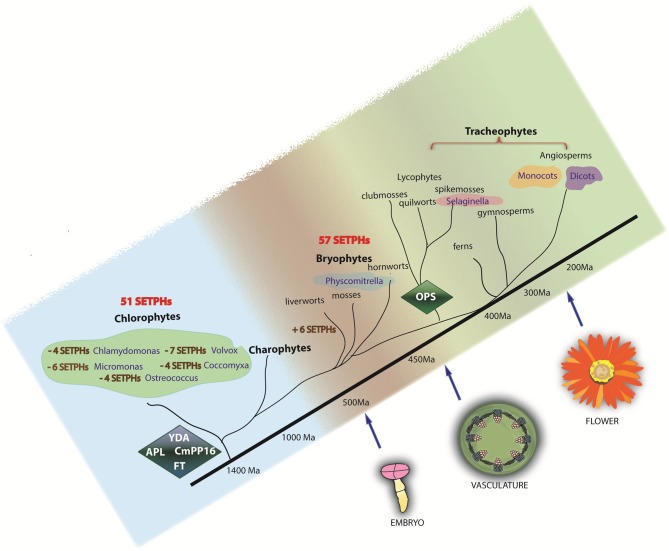
**Phylogeny of SETPHs in plants**. Presence of SETPHs are shown in each taxa. Minus signs refer to absence of orthologs in chlorophytes. *YDA*, *CmPP16*, *FT* and *APL* are present in chlorophytes and thus can be considered as ancient genes while *OPS* likely appeared first in *Selaginella* and is thus considered an innovation.

Besides a strict coordination of the activity of SETPHs, it is likely that the recruitment of factors already present in the tracheophytes ancestor, as well as the appearance of novel ones (for example via editing, alternative splicing, genome duplication and/or gene rearrangements) was important for the evolution of the plant vascular system. Our results involving the phylogenetic analysis of selected proteins required for phloem differentiation and/or function (such as long-distance signaling) support such notion (Figure 5).

Many important genes involved in land plant development regulation are highly conserved such as transcription factors gene families. Most are shared among angiosperms, and mosses and only a few are present in chlorophytes (Riaño-Pachón et al., 2008). APL was one of the first proteins identified involved in phloem differentiation; as such, no close homologs were expected in chlorophytes. However, APL is a member of the MYB transcription factor family, which regulate cell proliferation and differentiation in distantly related taxa from animals to fungi (Prouse and Campbell, 2012), proteins containing a MYB domain were actually found in the SETPHs of chlorophytes, but the similarity to tracheophyte APL is limited precisely to the MYB domain. The ancient tracheophyte *S. moellendorfii* forms a monophyletic group with *P. patens* as a sister taxon (Figure S1). A possible explanation could be that APL function in *S. moellendorfii* is more related to its role in *P. patens* predating its recruitment in sieve element and companion cell differentiation in more recent lineages. The existence of this protein in algae and non-vascular plants suggests a different role in ancestral lineages and its later recruitment as a part of the phloem development machinery.

APL thus originated from a preexisting protein, involved in the transcriptional regulation of a set of genes. Since the DNA-binding domain of the MYB proteins is similar, it is reasonable to assume that the target genes may be similar in chlorophytes and embryophytes. Additionally, these proteins show a continuum in sequence similarity, when *S. moellendorffii* and *P. patens* are considered, throughout all plant taxa. Also, MYB factors regulate a number of processes, including secondary metabolism, cell proliferation and differentiation and response to environmental stress (Dubos et al., 2010) some of which are likely absent in chlorophytes, so this recruitment of preexisting factors must have occurred several times during plant evolution.

OCTOPUS is an example of a protein factor involved in phloem differentiation that arose *de novo*. As described in the previous section, no homologs for this protein were found in chlorophytes. The groupings do not reflect the established phylogenetic relationships between tracheophytes, and unequal length of branches is evident, suggesting a rapid evolution of this gene, as well as adaptation of given taxa to particular niches. Its functional similarity with XYLOGEN raises the question about how two different proteins not related by ancestry could display similar functions in vascular tissue ontogeny (Truernit et al., 2012). This question cannot be answered satisfactorily based solely on bioinformatic analysis; experimental studies involving overexpression and deletion of this gene will be required to elucidate this functional similarity.

Flower induction has been known for decades to involve long distance communication through the phloem (Jaeger et al., 2006; Corbesier et al., 2007; Lin et al., 2007; Tamaki et al., 2007). Flowering locus T (FT) is a strong candidate for being the florigenic signal, although a role for its RNA cannot be discarded.

Evolutionary analysis of streptophytes, which include the basal land plants and charophyte algae, indicate that features such as plasmodesmata and apical growth possibly appeared before land colonization by plants (Becker and Marin, 2009). Furthermore, the presence of mobile proteins such as members of the KNOX protein family in chlorophyte algae indicates that plasmodesmal function is evolutionarily conserved (Pires and Dolan, 2012). It is therefore possible that the intercellular transport of the FT ancestor occurs in streptophytes, but evidently serving a different function.

The phylogenetic tree for FT (Figure S3) shows that this protein is present in *P. patens* and *S. moellendorffii*; it is probable that FT had rudimentary functions that resemble its role in angiosperms, involving cell to cell movement conceivably required for controlling apical growth. No FT homologs exist in chlorophytes, with the exception of *C. subellipsoidea* (two potential homologs present in the Phytozome genome database), which was used as outgroup. Two bursts of evolutionary change occurred in the lineages giving rise to *P. patens and S. moellendorffii.* One important burst is also observed within the angiosperms in the monocot clade, which is in agreement with the number of potential protein homologs clustered within this subgroup; no other important changes in the evolutionary rate are evident within angiosperms. One explanation for this could be that no important duplication events occurred during the evolution of *FT* genes in flowering plants outside the monocots. Three major clades are observed in the unrooted tree for flowering plants (Figure S6); two different clades in dicots, one of them being the most conserved, and monocots showing the most substitutions from the ancestral FT. *V. vinifera* was grouped alone and therefore it was not possible to infer its real relationship with other taxa.

Studies of viral movement proteins in plant systems have highlighted important mechanisms used for plant virus to utilize the host machinery operating in long distance communication system in plants and hence establish viral infection (Gilbertson and Lucas, 1996; Simón-Buela and García-Arenal, 1999; Lough and Lucas, 2006; Requena et al., 2006). CmPP16 is a plant paralog of viral movement proteins and is likewise capable to bind RNA in a non-selective manner and to interact and modify plasmodesmatal size exclusion limit (SEL) (Xoconostle-Cázares et al., 1999). Although much effort has been done to understand plant virus evolution, its rapid change rate difficults a better understanding of the functional constraints operating in the elements, responsible for the selective transport of macromolecules through the phloem long-distance communication system. A phylogenetic analysis of this protein was carried out using the Maximum Likelihood method using *O. lucimarinus* as the outgroup, a dendrogram is shown with considerably variation rates among all the taxa under study with no easily distinguishable natural clades beyond Brassicaceae (resolved with values of bootstrap >70%).

In plants the MAPK protein family is quite extensive, compared to mammals and other organisms. Only in Arabidopsis there are 20 genes encoding MAPKs, 10 MAPKKs and 60 MAPKKKs, whereas in yeast there are six for each [MAPK Group (Mitogen-activated protein kinases), 2002]. One of these proteins is the Arabidopsis protein YDA (a MAPKKK), which controls the asymmetric cell division in stomata, embryo development and inflorescence architecture (Bergmann et al., 2004; Lukowitz et al., 2004; Meng et al., 2012). Experimental analysis of the *YDA* gene promoter showed that it is not only active in stomata but also in companion cells (Ruiz-Medrano et al., 2011). Since phloem differentiation involves the asymmetric division of a phloem mother cell, phloem expression of this gene suggests a role for YDA in the development of this tissue.

In addition to YDA, the stomatal and embryo developmental programs share other elements of the MAPK pathway. These tissues predate the origin of the vascular tissue; therefore it can be hypothesized that YDA as well as other factors could have been recruited during the appearance of the vascular tissue (Figure 5).

## Concluding remarks

The plant vascular system allowed the great diversification of extant plants, in geographical distribution, structural diversity and size. The fossil record has shed light on the morphological steps likely to have occurred during the evolution of this tissue. The analysis of the genes involved in vascular tissue cell differentiation in different species with varying degree of vascular specialization, or lack thereof may help reconstruct which molecular factors were initially required for the appearance of the vasculature. On the other hand, the analysis of the expression pattern of this set of genes in both vascular and non-vascular plants will be important to reconstruct the genetic networks involved in its evolution. Given the importance of phloem and xylem in plant productivity (and that most, if not all, crops are tracheophytes), this knowledge will have important applications. A more complete knowledge of such networks will benefit from the deciphering of gymnosperm genomes, as well as from streptophytes, the closest algal relatives of land plants. In all, our results suggest that the coordinate expression of a set of genes, as well as the appearance of novel genes, and the recruitment of existing ones with a role in other signaling and developmental networks, were important for the evolution of the plant vascular system.

### Conflict of interest statement

The authors declare that the research was conducted in the absence of any commercial or financial relationships that could be construed as a potential conflict of interest.

## Supplementary material

Table S1 **Types of Motifs and frequency of Motifs in analyzed SETPHs**. Alignments in FASTA format: APL, OPS, FT, CmPP16, YDA.

Figure S1 **Phylogenetic analysis of the *APL* gene**. Arabidopsis APL protein sequence was retrieved form TAIR (http://www.arabidopsis.org) and used as query. Protein homologs were identified by BLAST search against the phytozome database (http://phytozome.net). Full length protein sequences were aligned with ClustalX2. The alignment parameters used were: Pairwise alignment: Gap opening 35, Gap extension 0.7; Multiple alignment: Gap opening 15, Gap extension 0.3. Evolutionary analyses were conducted in MEGA5. The evolutionary Phylogenetic reconstruction (shown as dendrogram) was inferred using the maximum likelihood statistical method. Bootstrap values higher than 70% are shown (1000 replicates). The analysis involved 37 amino acid sequences. Chlorophytes were used as outgroups to improve the phylogenetic tree. Red triangle: Dicots, Blue triangle: monocots, Purple triangle: lycophytes. Green square: Bryophytes, Gray circle: Chlorophytes. Species and accession number are shown. The same symbols were used in subsequent figures.

Figure S2 **Phylogenetic analysis of FT gene in plant species**. Arabidopsis FT protein sequence was retrieved form TAIR (http://www.arabidopsis.org) and used as query. Protein homologs were identified by BLAST search against the phytozome database (http://phytozome.net). Full length protein sequences were aligned with ClustalX2. The alignment parameters used were: Pairwise alignment: Gap opening 35, Gap extension 0.7; Multiple alignment: Gap opening 15, Gap extension 0.3. Evolutionary analyses were conducted in MEGA5. The evolutionary Phylogenetic reconstruction (shown as dendrogram) was inferred using the maximum likelihood statistical method. Bootstrap values higher than 70% are shown (1000 replicates). The analysis involved 35 amino acid sequences. *C. subellipsoidea* was used as outgroup.

Figure S3 **Phylogenetic analysis of OCTOPUS gene in plant species**. Arabidopsis OCTOPUS protein sequence was retrieved form TAIR (http://www.arabidopsis.org) and used as query. Protein homologs were identified by BLAST search against the phytozome database (http://phytozome.net). Full length protein sequences were aligned with ClustalX2. The alignment parameters used were: Pairwise alignment: Gap opening 35, Gap extension 0.7; Multiple alignment: Gap opening 15, Gap extension 0.3. Evolutionary analyses were conducted in MEGA5. The evolutionary Phylogenetic reconstruction (shown as dendrogram) was inferred using the neighbor-joining statistical method. Bootstrap values higher than 70% are shown (1000 replicates). The analysis involved 30 amino acid sequences. *S. moellendorffii* was used as outgroup.

Figure S4 **Phylogenetic analysis of CmPP16 homologs**. CmPP16 protein sequence was retrieved form TAIR (http://www.arabidopsis.org) and used as query. Protein homologs were identified by BLAST search against the phytozome database (http://phytozome.net). Full-length protein sequences were aligned with ClustalX2. The alignment parameters used were: Pairwise alignment: Gap opening 35, Gap extension 0.7; Multiple alignment: Gap opening 15, Gap extension 0.3. Evolutionary analyses were conducted in MEGA5. The evolutionary Phylogenetic reconstruction (shown as dendrogram) was inferred using the maximum likelihood statistical method. Bootstrap values higher than 70% are shown (1000 replicates). The analysis involved 37 amino acid sequences. *C. subellipsoidea* was used as outgroup.

Figure S5 **Phylogenetic analysis of YDA protein**. The Arabidopsis YDA protein sequence was retrieved form TAIR (http://www.arabidopsis.org) and used as query. Protein homologs were identified by BLAST search against the phytozome database (http://phytozome.net). Full length protein sequences were aligned with ClustalX2. The alignment parameters used were: Pairwise alignment: Gap opening 35, Gap extension 0.7; Multiple alignment: Gap opening 15, Gap extension 0.3. Evolutionary analyses were conducted in MEGA5. The evolutionary Phylogenetic reconstruction (shown as dendrogram) was inferred using the neighbor-joining statistical method. Bootstrap values higher than 70% are shown (1000 replicates). The analysis involved 39 amino acid sequences. *C. subellipsoidea* was used as outgroup.

Figure S6 **FT unrooted tree**. Full length protein sequences were aligned with ClustalX2. The alignment parameters used were: Pairwise alignment: Gap opening 35, Gap extension 0.7; Multiple alignment: Gap opening 15, Gap extension 0.3. Evolutionary analyses were conducted in MEGA5. The evolutionary Phylogenetic reconstruction was inferred using the maximum likelihood statistical method. Bootstrap values higher than 70% are shown (1000 replicates).

## References

[B1] Bailey T. L. Elkan C. (1994). Fitting a mixture model by expectation maximization to discover motifs in biopolymers, in Proceedings of the Second International Conference on Intelligent Systems for Molecular Biology, (Menlo Park, CA: AAAI Press), 28–36 7584402

[B2] Banks J. A. Nishiyama T. Hasebe T. Bowman J. M. Gribskov M. dePamphilis C. (2011). The Selaginella genome identifies genetic changes associated with the evolution of vascular plants. Science 332, 960–963 10.1126/science.1203810 21551031 PMC3166216

[B3] Becker B. Marin B. (2009). Streptophyte algae and the origin of embryophytes. Ann. Bot. 103, 999–1004 10.1093/aob/mcp044 19273476 PMC2707909

[B4] Bergmann D. Lukowitz W. Somerville C. (2004). Stomatal development and pattern controlled by a MAPKK kinase. Science 304, 1494–1497 10.1126/science.1096014 15178800

[B5] Bonke M. Thitamadee S. Mähönen A. Hauser M.-T. Helariutta Y. (2003). APL regulates vascular tissue identity in Arabidopsis. Nature 426, 181–186 10.1038/nature02100 14614507

[B6] Burr F. A. Evert R. F. (1973). Some aspects of sieve-element structure and development in Selaginella kraussiana. Protoplasma 78, 81–97 10.1007/BF01281524

[B7] Corbesier L. Vincent C. Jang S. Fornara F. Fan Q. Searle L. (2007). FT Protein movement contributes to long-distance signaling in floral induction in arabidopsis. Science 316, 1030–1036 10.1126/science.1141752 17446353

[B8] Crooks G. E. Hon G. Chandonia J. M. Brenner S. E. (2004). WebLogo: a sequence logo generator. Genome Res. 14, 1188–1190 10.1101/gr.849004 15173120 PMC419797

[B9] Dubos C. Stracke R. Grotewold E. Weisshaar B. Martin C. Lepiniec L. (2010). MYB transcription factors in Arabidopsis. Trends Plant Sci. 15, 573–581 10.1016/j.tplants.2010.06.005 20674465

[B10] Gilbertson R. L. Lucas W. J. (1996). How do viruses traffic on the vascular higway? Trends Plant Sci. 1, 260–268 10.1016/1360-1385(96)10029-7

[B11] Gouy M. Guindon S. Gascuel O. (2010). SeaView version 4: a multiplatform graphical user interface for sequence alignment and phylogenetic tree building. Mol. Biol. Evol. 27, 221–224 10.1093/molbev/msp259 19854763

[B12] Hamel L.-P. Nicole M.-C. Sritubtim S. Morency M.-J. Ellis M. Ehlting J. (2006). Ancient signals: comparative genomics of plant MAPK and MAPKK gene families. Trends Plant Sci. 11, 192–198 10.1016/j.tplants.2006.02.007 16537113

[B13] Harmer S. L. Hogenesch J. B. Straume M. Chang H. S. Han B. Zhu T. (2000). Orchestrated transcription of key pathways in Arabidopsis by the circadian clock. Science 290, 2110–2113 10.1126/science.290.5499.2110 11118138

[B14] Jaeger K. E. Graf A. Wigge P. A. (2006). The control of flowering in time and space. J. Exp. Bot. 57, 3415–3418 10.1093/jxb/erl159 17005922

[B15] Kardailsky I. Shukla V. K. Ahn J. H. Dagenais N. Christensen S. K. Nguyen J. T. (1999). Activation tagging of the floral inducer FT. Science 286, 1962–1965 10.1126/science.286.5446.1962 10583961

[B16] Krusell L. Madsen L. H. Sato S. Aubert G. Genua A. Szczyglowski K. (2002). Shoot control of root development and nodulation is mediated by a receptor-like kinase. Nature 420, 422–426 10.1038/nature01207 12442170

[B17] Larkin M. A. Blackshields G. Brown N. P. Chenna R. McGettigan P. A. McWilliam H. (2007). Clustal W and Clustal X version 2.0. Bioinformatics 23, 2947–2948 10.1093/bioinformatics/btm404 17846036

[B18] Lata C. Prasad M. (2011). Role of DREBs in regulation of abiotic stress responses in plants. J. Exp. Bot. 62, 4731–4748 10.1093/jxb/err210 21737415

[B19] Li Y. Liu Z. B. Shi X. Hagen G. Guilfoyle T. J. (1994). An auxin-inducible element in soybean SAUR promoters. Plant Physiol. 106, 37–43 10.1104/pp.106.1.37 7972520 PMC159496

[B20] Li X. Wu H. X. Southerton S. G. (2010). Comparative genomics reveals conservative evolution of the xylem transcriptome in vascular plants. BMC Evol. Biol. 10:190 10.1186/1471-2148-10-190 20565927 PMC2907377

[B21] Lin M. K. Belanger H. Lee Y. J. Varkonyi-Gasic E. Taoka K. Miura E. (2007). FLOWERING LOCUS T protein may act as the long-distance florigenic signal in the cucurbits. Plant Cell 19, 1488–1506 10.1105/tpc.107.051920 17540715 PMC1913722

[B22] Liu H. Yu X. Li K. Klejnot J. Yang H. Lisiero D. (2008). Photoexcited CRY2 interacts with CIB1 to regulate transcription and floral initiation in Arabidopsis. Science 322, 1535–1539 10.1126/science.1163927 18988809

[B23] Lough T. J. Lucas W. J. (2006). Integrative plant biology: role of phloem long- distance macromolecular trafficking. Annu. Rev. Plant Biol. 57, 203–232 10.1146/annurev.arplant.56.032604.144145 16669761

[B24] Lucas W. J. Groover A. Lichtenberger R. Furuta K. Yadav S. R. Helariutta Y. (2013). The Plant Vascular System: evolution, development and functions. J. Integr. Plant Biol. 10.1111/jipb.12041 23462277

[B25] Lukowitz W. Roeder A. Parmenter D. Somerville C. (2004). A MAPKK kinase gene regulates extra-embryonic cell fate in Arabidopsis. Cell 116, 109–119 10.1016/S0092-8674(03)01067-5 14718171

[B26] MAPK Group (Mitogen-activated protein kinases). (2002). Mitogen-activated protein kinase cascades in plants: a new nomenclature. Trends Plant Sci. 7, 301–308 10.1016/S1360-1385(02)02302-6 12119167

[B27] Meng X. Wang H. He Y. Liu Y. Walker J. Torii K. (2012). A MAPK cascade downstream of ERECTA receptor-like protein kinase regulates Arabidopsis inflorescence architecture by promoting localized cell proliferation. Plant Cell 24, 4948–4960 10.1105/tpc.112.104695 23263767 PMC3556968

[B28] Nei M. Kumar S. (2000). Molecular Evolution and Phylogenetics. New York, NY: Oxford University Press

[B29] Niklas K. J. (1997). The Evolutionary Biology of Plants. Chicago, IL: University of Chicago Press

[B30] Ottesen E. A. Young C. R. Eppley J. M. Ryan J. P. Chavez F. P. Scholin C. A. (2013). Pattern and synchrony of gene expression among sympatric marine microbial populations. Proc. Natl. Acad. Sci. U.S.A. 110, E488–E497 10.1073/pnas.1222099110 23345438 PMC3568374

[B31] Pires N. Dolan L. (2012). Morphological evolution in land plants: new designs with old genes. Phil. Trans. R. Soc. Lond. B Biol. Sci. 367, 508–518 10.1098/rstb.2011.0252 22232763 PMC3248709

[B32] Prouse M. B. Campbell M. M. (2012). The interaction between MYB proteins and their target DNA binding sites. Biochim. Biophys. Acta 1819, 67–77 10.1016/j.bbagrm.2011.10.010 22067744

[B33] Requena A. Simón-Buela L. Salcedo G. García-Arenal F. (2006). Potential involvement of a cucumber of phloem protein 1 in the long distance movement of cucumber mosaic virus particles. Mol. Plant Microbe Interact. 19, 734–746 10.1094/MPMI-19-0734 16838786

[B34] Riaño-Pachón D. Corrêa L. G. Trejos-Espinosa R. l. Mueller-Roeber B. (2008). Green transcription factors: a chlamydomonas overview. Genetics 179, 31–39 18493038 10.1534/genetics.107.086090 PMC2390610

[B35] Ruiz-Medrano R. Guevara-González R. G. Argüello-Astorga G. R. Monsalve- Fonnegra Z. Herrera-Estrella L. R. Rivera-Bustamante R. F. (1999a). Identification of a sequence element involved in AC2-mediated transactivation of the pepper huasteco virus coat protein gene. Virology 253, 162–169 10.1006/viro.1998.9484 9918875

[B36] Ruiz-Medrano R. Xoconostle-Cázares B. Lucas W. J. (1999b). Phloem long- distance transport of CmNACP mRNA: implications for supracellular regulation in plants. Development 126, 4405–4419 10498677 10.1242/dev.126.20.4405

[B37] Ruiz-Medrano R. Hinojosa-Moya J. J. Xoconostle-Cázares B. Lucas W. J. (2007). Influence of Cucumber mosaic virus infection on the mRNA population present in the phloem translocation stream of pumpkin plants. Funct. Plant Biol. 34, 292–301 10.1071/FP06300 32689355

[B38] Ruiz-Medrano R. Xoconostle-Cázares B. Ham B.-K. Li G. Lucas W. J. (2011). Vascular expression in Arabidopsis is predicted by the frequency of CT/GA-rich repeats in gene promoters. Plant J. 67, 130–144 10.1111/j.1365-313X.2011.04581.x 21435051

[B39] Santi L. Wang Y. Stile M. R. Berendzen K. Wanke D. Roig C. (2003). The GA octodinucleotide repeat binding factor BBR participates in the transcriptional regulation of the homeobox geneBKn3. Plant J. 34, 813–826 10.1046/j.1365-313X.2003.01767.x 12795701

[B40] Sauter A. Davies W. J. Hartung W. (2001). The long-distance abscisic acid signal in the droughted plant: the fate of the hormone on its way from root to shoot. J. Exp. Bot. 52, 1991–1199 10.1093/jexbot/52.363.1991 11559734

[B41] Schneidereit A. Imlau A. Sauer N. (2008). Conserved cis-regulatory elementsfor DNA-binding-with-one-finger and homeo-domain-leucine-zipper transcription factors regulate companion cell-specific expression of the *Arabidopsis thaliana SUCROSE TRANSPORTER 2* gene. Planta 228, 651–662 10.1007/s00425-008-0767-4 18551303

[B42] Schwarz R. Dayhoff M. (1979). Matrices for detecting distant relationships, in Atlas of Protein Sequences, ed Dayhoff M. (National Biomedical Research Foundation), 353–358

[B42a] Simón-Buela L. García-Arenal F. (1999). Virus particles of cucumber green mottle mosaic tobamovirus move systemically in the phloem of infected cucumber plants. Mol. Plant Microbe Interact. 12, 112–118 10.1094/MPMI.1999.12.2.112 9926413

[B44] Sunter G. Bisaro D. M. (1997). Regulation of a Geminivirus coat protein promoter by AL2 Protein (TrAP): evidence for activation and derepression mechanisms. Virology 232, 269–280 10.1006/viro.1997.8549 9191840

[B45] Tamaki S. Matsuo S. Wong H. L. Yokoi S. Shimamoto K. (2007). Hd3a protein is a mobile flowering signal in rice. Science 316, 1033–1036 10.1126/science.1141753 17446351

[B46] Tamura K. Peterson D. Peterson N. Stecher G. Nei M. Kumar S. (2011). MEGA5: molecular evolutionary genetics analysis using maximum likelihood, evolutionary distance, and maximum parsimony methods. Mol. Biol. Evol. 28, 2731–2739 10.1093/molbev/msr121 21546353 PMC3203626

[B46a] Taoka K.-i. Ohki I. Tsuji H. Furuita K. Hayashi K. Yanase T. (2011). 14-3-3 proteins act as intracellular receptors for rice Hd3a florigen. Nature 476, 332–335 10.1038/nature10272 21804566

[B47] Truernit E. Bauby H. L. N. Belcram K. Barthélémy J. Palauqui J.-C. (2012). OCTOPUS, a polarly localised membrane-associated protein, regulates phloem differentiation entry in *Arabidopsis thaliana*. Development 139, 1306–1315 10.1242/dev.072629 22395740

[B48] Xoconostle-Cázares B. Xiang Y. Ruiz-Medrano R. Wang H. L. Monzer J. Yoo B. C. (1999). Plant paralog to viral movement protein that potentiates transport of mRNA into the phloem. Science 283, 94–98 10.1126/science.283.5398.94 9872750

[B49] Zwack P. J. Shi X. Robinson B. R. Grupta S. Compton M. A. Gerken D. M. (2012). Plant Cell Physiol. 53, 1683–1695 10.1093/pcp/pcs110 22864451

