# Supplementary material for: Vascular gene expression: a hypothesis
Source: Front Plant Sci. 2013 Jul 17;4:261. doi: 10.3389/fpls.2013.00261 (PMC3713349; doi:10.3389/fpls.2013.00261)

ALTERED PHLOEM DEVELOPMENT (APL)

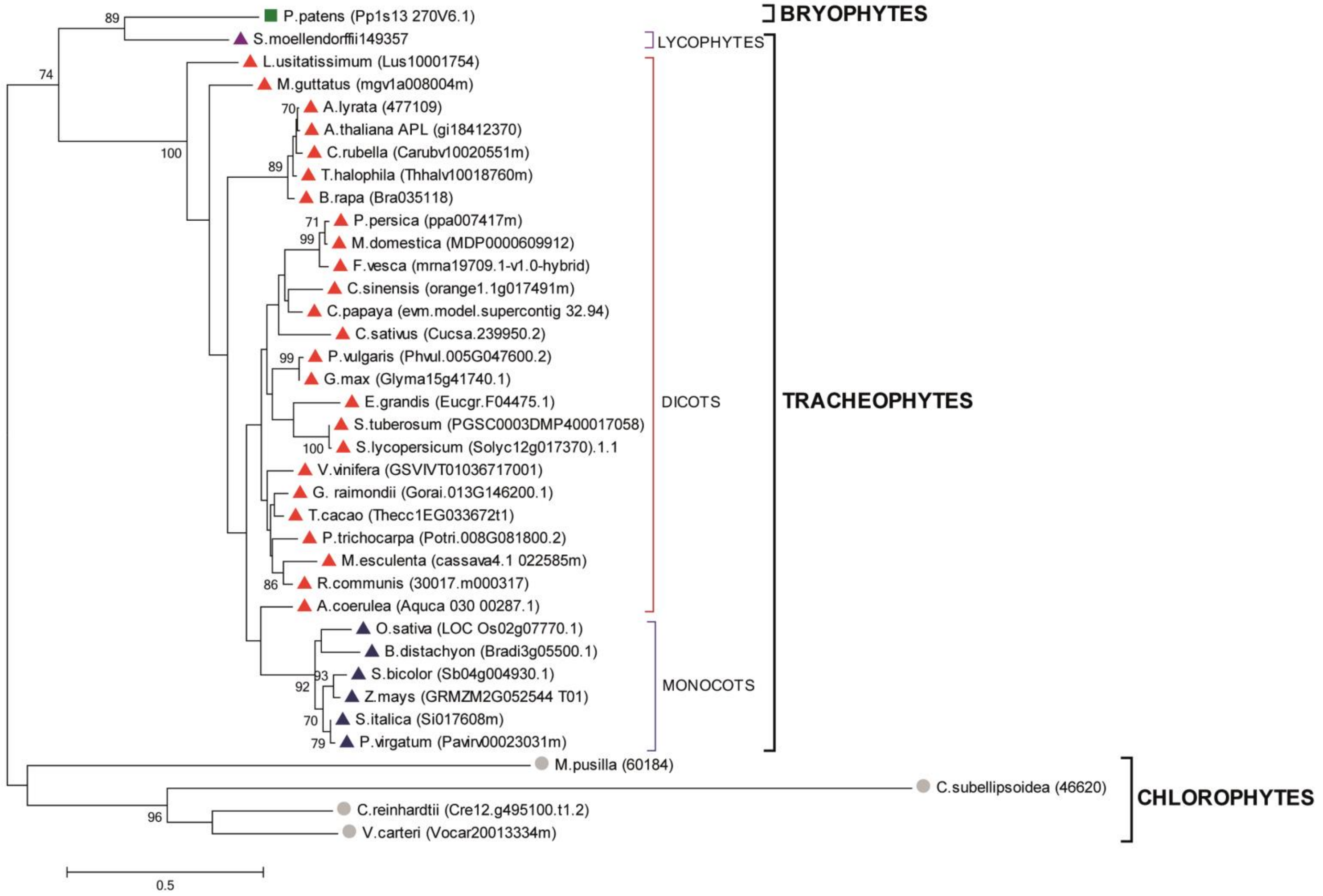

Supplementary Figure 1
Martínez-Navarro *et al.*, 2013

Supplementary Figure 2
Martínez-Navarro *et al.*, 2013

OCTOPUS (OPS)

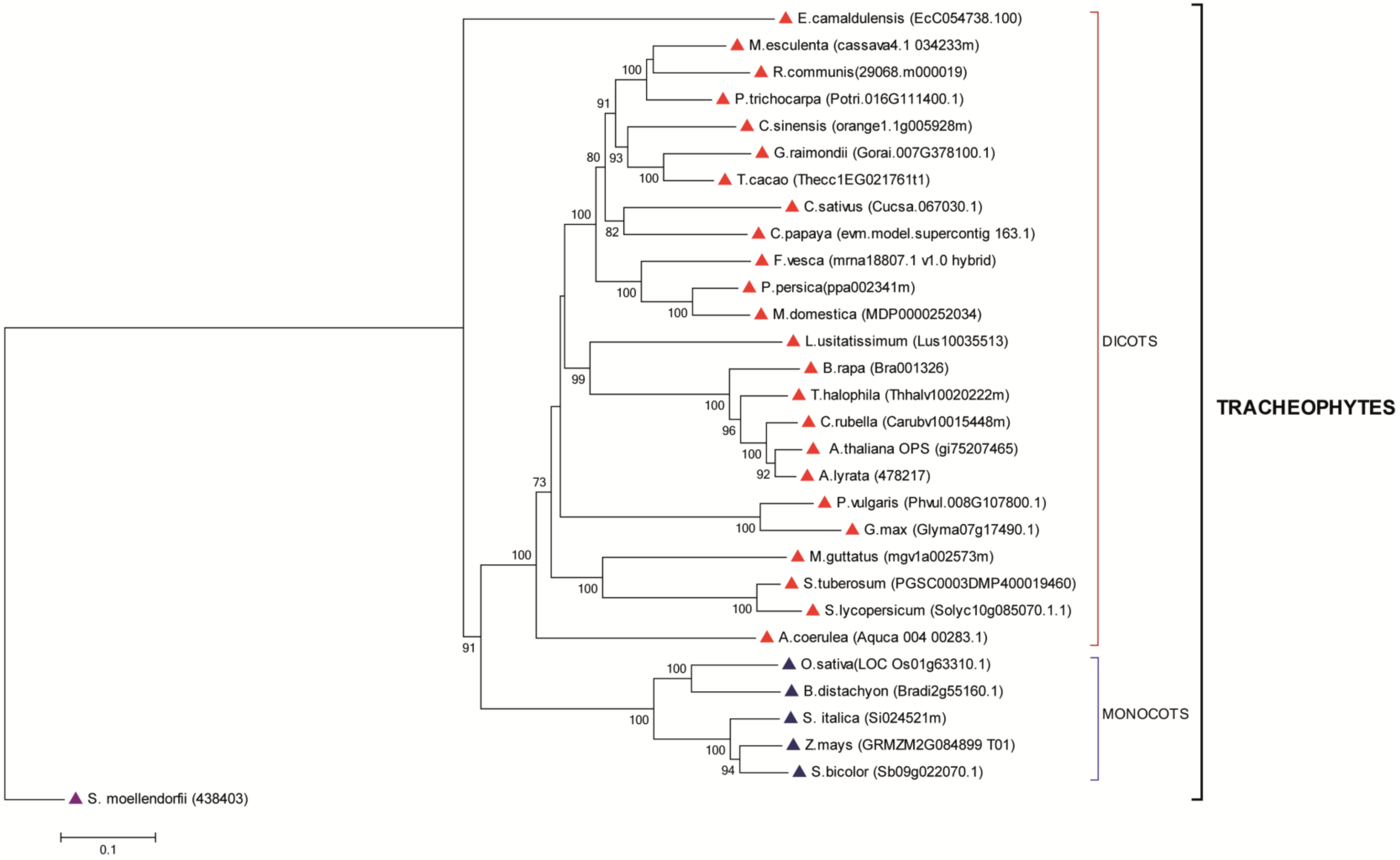

FLOWERING LOCUS T (FT)

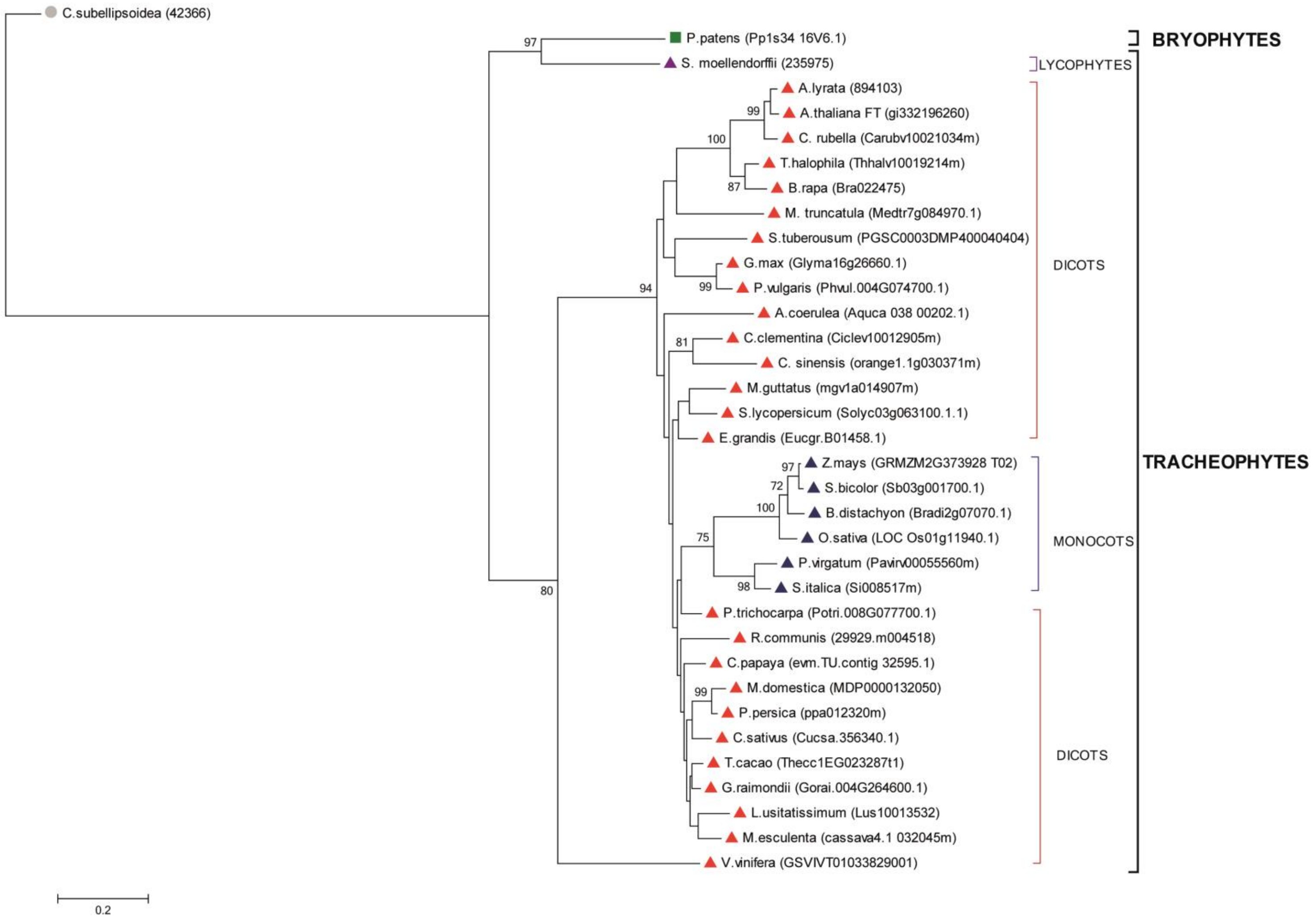

Supplementary Figure 3
Martínez-Navarro *et al.*, 2013

CmPP16

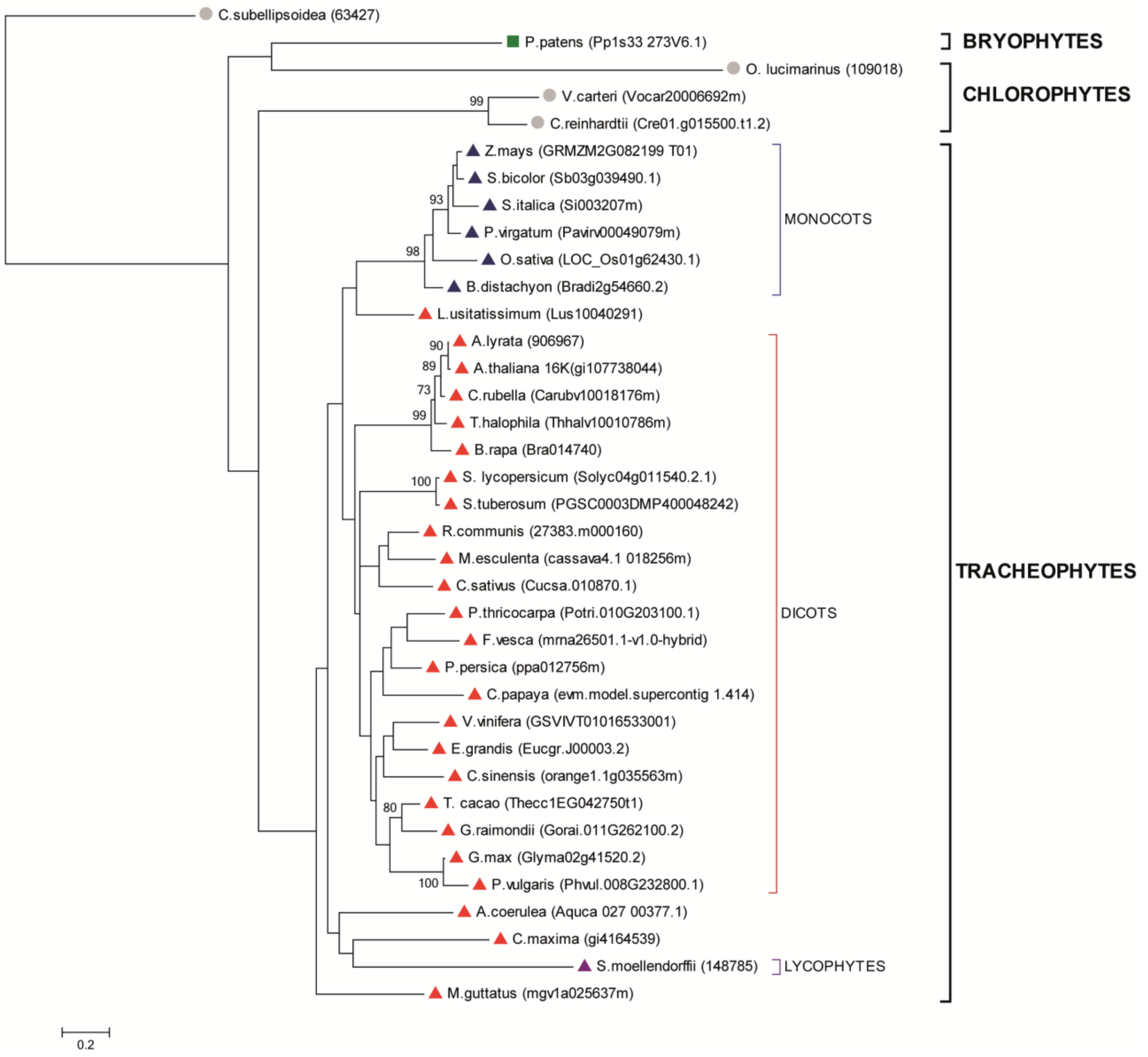

Suppleentary Figure 4
Martínez-Navarro *et al.*, 2013

YDA

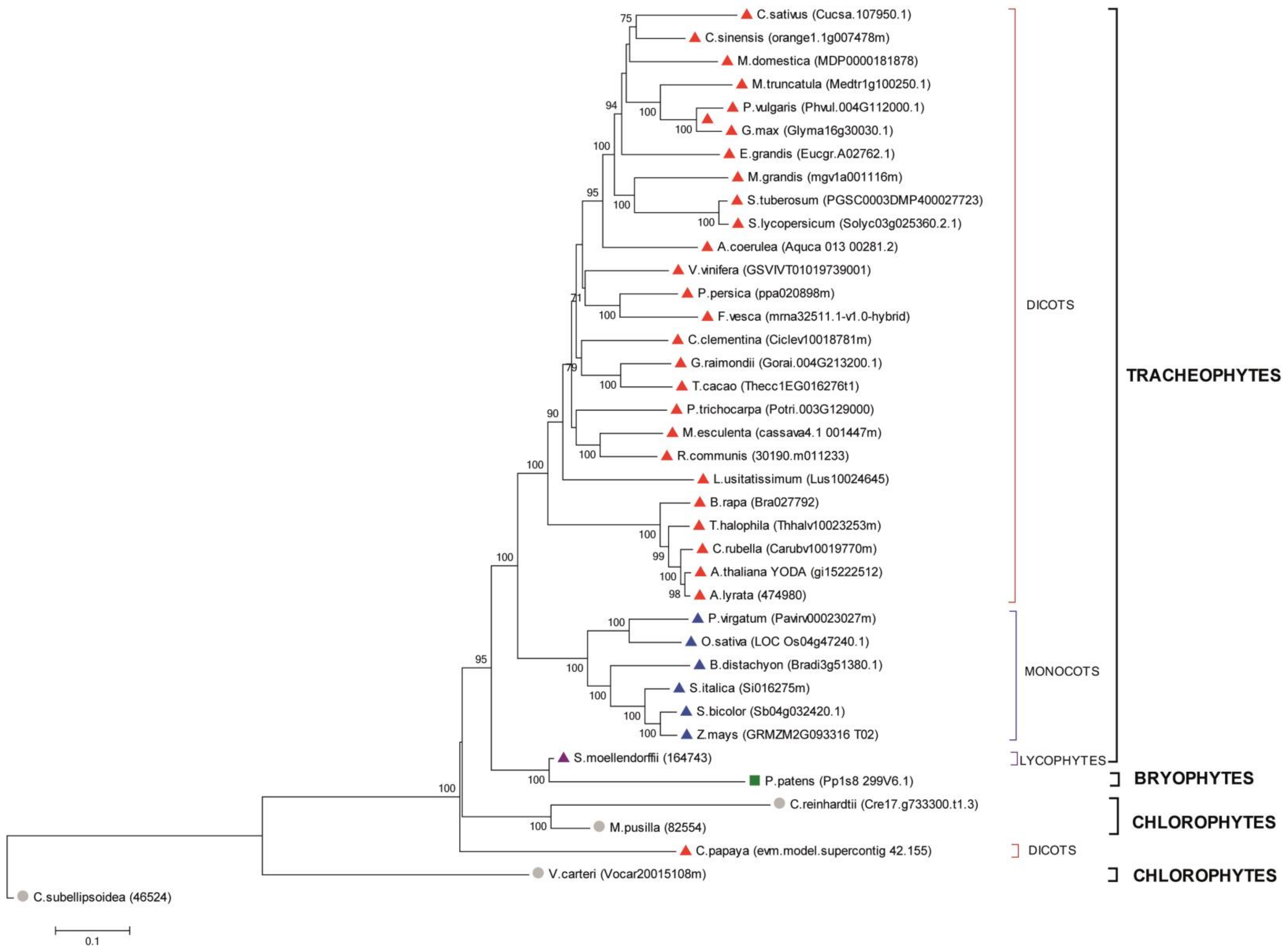

Supplemetary Figure 5
Martínez-Navarro *et al.*, 2013

Supplementary Figure 6
Martínez-Navarro *et al.*, 2013

FLOWERING LOCUS T

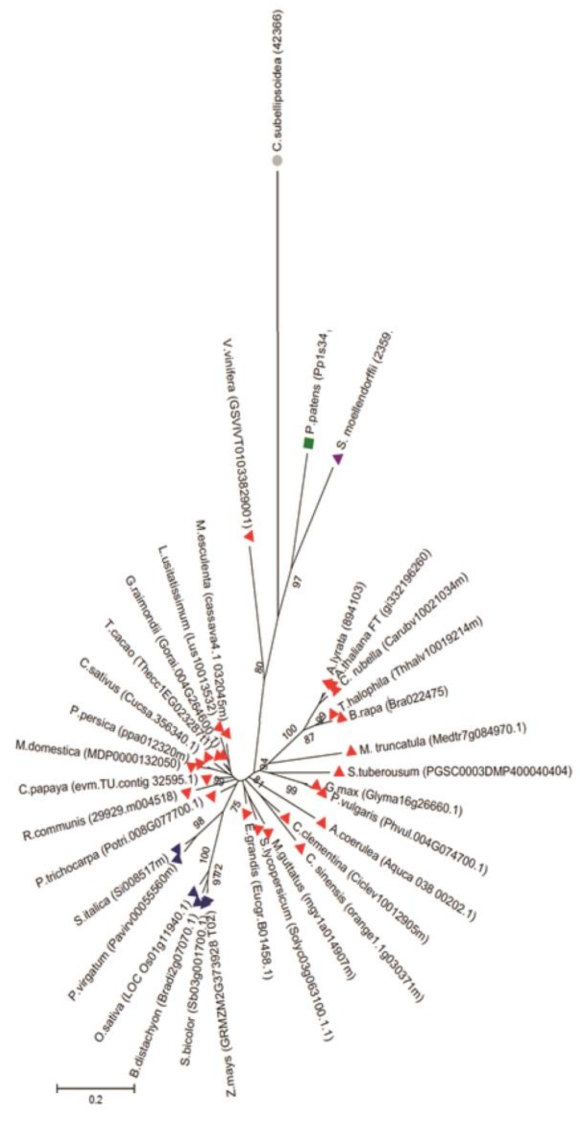

Supplement: Supplementary file 8 [file 52375_Ruiz-Medrano_Presentation1.PDF]
